# Preoperative radiologic predictors of successful soft tissue release surgery for hip subluxation among cerebral palsy patients: A STROBE compliant study

**DOI:** 10.1097/MD.0000000000011847

**Published:** 2018-08-17

**Authors:** Myongsu Ha, Takashi Okamoto, Toshitsugu Fukuta, Yoshiaki Tsuboi, Yasuhiro Shirai, Kazuki Hattori, Eisuke Sakuma, Kenjiro Wakabayashi, Ikuo Wada, Takanobu Otsuka

**Affiliations:** a Department of Orthopedic Surgery; b Department of Molecular and Cellular Biology; c Department of Integrative Anatomy; d Department of Rehabilitation Medicine, Nagoya City University Graduate School of Medical Sciences, Nagoya, Japan.

**Keywords:** acetabular index, cerebral palsy, hip subluxation, soft tissue release surgery

## Abstract

Paralytic hip subluxation is a common problem in children with cerebral palsy. Although surgical procedures such as soft tissue release and osteotomy have been advocated for its prevention, the exact indications of such procedures remain unclear. We attempted to evaluate preoperative radiographic parameters and identify prognostic factors in children with cerebral palsy. We retrospectively investigated 43 hips in 27 children with cerebral palsy who had undergone soft tissue release surgery for hip subluxation. We evaluated the age at the time of surgery and the radiographic parameters such as the center-edge angle (CEA), the migration percentage (MP), and the acetabular index (AI) at 3 time points: preoperation, 1 year after surgery, and at final follow-up. The outcome measure was determined by the MP value at final follow-up. Student *t* test was used to compare the quantitative variables between 2 groups (good vs poor outcome). Then the multiple regression analysis was applied to determine the prognostic factors upon soft tissue release surgery. Children with good outcome exhibited higher CEA (average value of −1.43° vs −13.2° in those with poor outcome), lower MP (53.9% vs 71.3%), and lower AI (28.1° vs 35.3°). Upon multiple regression analysis, we found that the age at the time of surgery, preoperative CEA, and preoperative MP did not appear to be independent prognostic factors. The only independent factor that affected prognosis after soft tissue release surgery was the preoperative AI. The preoperative AI values <34° were associated with the good outcome with specificity of 87% and sensitivity of 60% according to the receiver operating characteristic curve analysis. These findings indicate that the outcome of soft tissue release surgery can be predicted by the preoperative AI value.

## 1 Introduction

Paralytic hip subluxation is a common cause for the impairment of the activities of daily living (ADL) in children with cerebral palsy. The orthopedic complications such as subluxation and dislocation of hip joints are the major medical concern for children with cerebral palsy.^[1–3]^

The reported incidence of subluxation or dislocation is quite variable and between 2% and 75%^[4]^ depending on the severity of cerebral palsy. The severity of hip subluxation and dislocation is associated with the extent of damage in the neuromuscular system such as spasticity and rigidity, and the risk of hip dislocation is closely correlated with gross motor function,^[5]^ leading to the physical inability for sitting and walking. Progressive hip subluxation and eventual dislocation are considered to be resulted from the muscle imbalance that is created by the stronger flexors and adductors associated with the weaker extensors and abductors.^[6]^ This muscle imbalance eventually leads to abnormal positioning of the hip joint during flexion, adduction, and internal rotation.

The extent of hip displacement has been measured in terms of the migration percentage (MP), as based on Reimers protocol described in 1980,^[7]^ in which it was suggested that the contracture of the adductor muscles is the primary cause of paralytic hip subluxation. He also demonstrated the gradual increase of MP, 10% increase every year, once the hip subluxation occurs.

Various surgical procedures such as soft tissue release and osseous surgeries have been advocated for the prevention of hip subluxation, mainly depending on its severity. The soft tissue release surgery (muscle dissections and muscle/tendon lengthening) have been proposed to correct the muscle imbalance, and proven beneficial in preventing the paralytic hip subluxation and dislocation. The surgical indications of soft tissue release are usually based on both the extent of displacement of femoral head and the severity of acetabular dysplasia. It has, however, not yet been clarified with regard to the indication for soft tissue release surgery. Thus, we have attempted to identify preoperative radiographic parameters being indicative for soft tissue release surgery for preventing paralytic hip subluxation with cerebral palsy.

In this study, we propose effective parameters by carefully examining various preoperative radiographic parameters. We also demonstrate the association of such parameters with surgical outcome of soft tissue release in children with cerebral palsy.

## 2 Methods

### 2.1 Patients and study design

This retrospective study was carried out with the ethical approval of the institutional review board of Nagoya City University, Nagoya (approval no.: 1297, issued on March 4, 2016). Oral informed consent was obtained from patients or his/her parents. The need for signing the informed consent was not obtained because of the retrospective nature of our study and no patient identification data were included in the analysis.

We reviewed patients with cerebral palsy who had undergone soft tissue release surgery between April 2004 and April 2015 in Nagoya City University Hospital.

### 2.2 Inclusion and exclusion criteria

In the aforementioned period, 32 patients with cerebral palsy had undergone soft tissue release surgery. We excluded the patients who could not be followed up <1 year and also omitted the children with insufficient clinical or radiographic information, or additional surgery performed case for dislocated hip. Two children were excluded with the problem of follow-up period, 2 children were excluded with the insufficient information, and the other 1 child had excluded because of the surgery for dislocated hip. Finally 27 children met the inclusion criteria.

### 2.3 Patients’ data

The mean age at the time of surgery was 4 years and 10 months (range, 2 years and 10 months to 10 years and 1 month). The mean follow-up period was 5 years and 5 months (range, 1 year and 1 month to 12 years).

Among the 27 patients studied, 25 patients belonged to the level V category in accordance with the preoperative Gross Motor Function Classification System (GMFCS)^[8]^ (Table 1), one each patient belonged to the level III and IV, but there was no patient belonged to the level I or II. In patients with unilateral subluxation, the healthy hip was excluded from the present analysis. Thus, the present analysis included 43 hips from 27 patients.

**Table 1:**
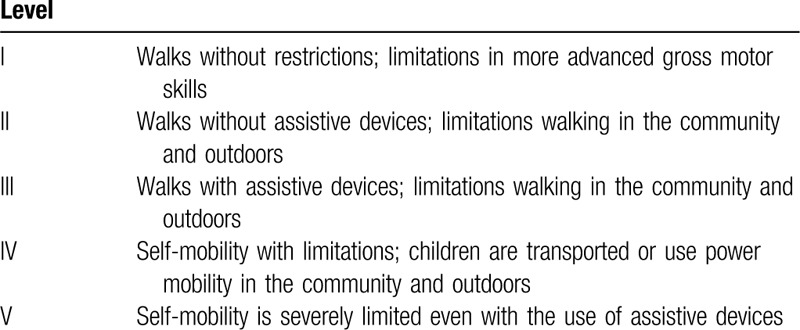
The Gross Motor Function Classification System.

### 2.4 Surgical procedures

In accordance with Reimers definition of subluxation,^[7]^ we performed soft tissue release surgery for those patients with MP value of >33%. Although cases with MP value of 100% were defined as dislocation, those with MP values between 33% and 99% were defined as having hip subluxation according Reimers definition.^[7]^ The following soft tissue release surgery was performed in 43 hips (27 patients): open adductor longus tenotomy and gracilis myotomy from the medial side; lengthening of rectus femoris muscle and iliopsoas tenotomy from the anterior side; and tenotomy of biceps femoris, semitendinosus muscle, and semimembranosus muscle from the posterior side. In addition, adductor magnus tenotomy was performed in 8 patients with knee joint contraction.

### 2.5 Postoperative treatment

A hip spica cast was applied and maintained for 2 to 4 weeks after surgery and then a hip abduction brace was used during remaining 6 months. After the hip spica casting has been removed, we conducted physical therapy and taught the methods of home exercise to the parents of patients for preventing postoperative joint contracture.

### 2.6 Measurements

**Figure 1:**
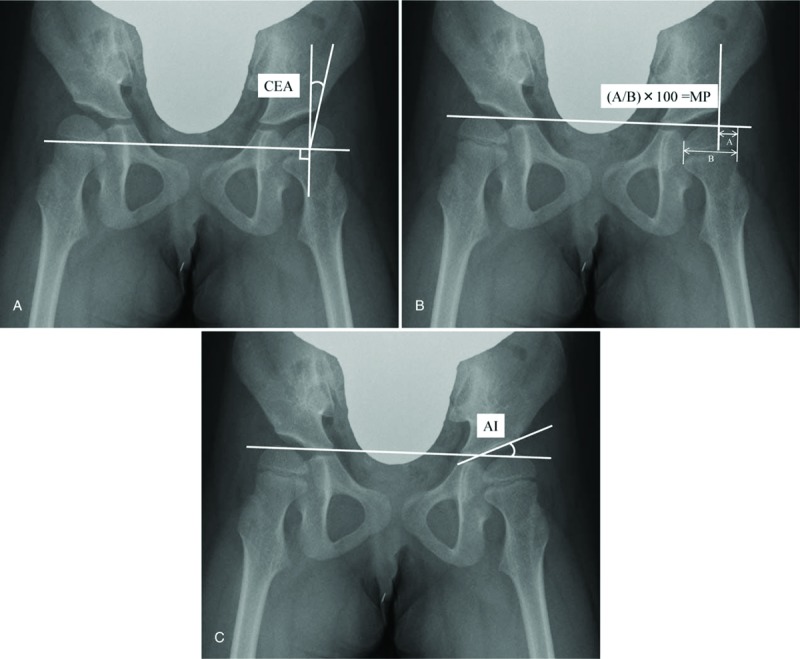
Radiological measurements. A, The center-edge angle (CEA) is the angle between the vertical line through the center of the femoral head and a line from the center through the lateral edge of the acetabular roof. B, Migration percentage (MP): the vertical lines (Perkins line) are drawn from the lateral edge of the acetabulum perpendicular to a horizontal line connecting both triradiate cartilages of the pelvis (Hilgenreiner line). The portion of the femoral head lateral to Perkins line is measured (A), and this measurement is expressed as a percentage of the entire horizontal width of the femoral head (B). C, Acetabular index (AI) is the angle between Hilgenreiner line and the line connecting the inferior medial and superolateral aspects of acetabulum.

The center-edge angle (CEA) was introduced by Wiberg^[9]^ as the measure of acetabular development and/or the degree of displacement of the femoral head. The CEA is defined as the angle that is formed between the vertical line (Perkins line) and a line drawn from the lateral edge of the acetabulum through the center of the femoral head (Fig. 1A).

The MP, initially described by Reimers,^[7]^ has been widely used in clinical practice to determine the radiographic degree of hip subluxation. In order to evaluate MP, Perkins lines were drawn from the lateral edge of the acetabulum perpendicular to the horizontal line connecting bilateral triradiate cartilages of the pelvis (Hilgenreiner line).^[10]^ The portion of the femoral head lateral to the Perkins line is measured (denoted as “A” in Fig. 1B), and is expressed as percentage of the entire horizontal width of the femoral head (“B” in Fig. 1B).

Measurement of the acetabular index (AI), described by Hilgenreiner,^[10]^ represents the conventional approach for evaluating acetabular dysplasia in young children with cerebral palsy. The AI is the angle between the Hilgenreiner line and the line connecting the inferomedial and superolateral aspects of the acetabulum (Fig. 1C). Normally, the AI is slightly <30° at birth and decreases to 22° at 1 year of age.^[11]^ AI measurement is considered crucial for the evaluation and the diagnosis of the acetabular dysplasia.^[12–14]^

In this study, all measurements were performed on anteroposterior radiographs of the hips, taken for the patient in supine position with the femora in neutral abduction-adduction position relative to the pelvis.

### 2.7 Outcome measures

In this study, the CEA, MP, and AI were measured from the radiographs of hip joints taken before the surgery, 1 year after surgery, and at final follow-up. To assess the intraobserver reliability, all radiographs were reviewed twice by the same investigator (HM). To assess the interobserver reliability, the same radiographs were reviewed by 2 investigators (HM and KH).

The patients were divided into 2 groups according to the MP value at final follow-up. A hip was considered to have good outcome if MP at final follow-up was <33%, and poor outcome if MP at final follow-up was ≥33%. The preoperative CEA, MP, and AI and the patient age at the time of surgery were evaluated in each group. We also evaluated the GMFCS level at final follow-up.

### 2.8 Statistical analysis

One-way analysis of variance (ANOVA) test was used to compare each radiographic parameter of all patients at the clinical stage of before surgery, 1 year after surgery and at final follow-up. The comparison of each radiographic parameter among different clinical stage was done using post-hoc test. Student *t* test was used to compare the radiographic parameters and the age between 2 groups (patients with either good or poor outcome). In order to identify independent prognostic factors, multiple regression analysis was performed with outcome (good, MP < 33%; poor, MP ≥ 33%) as the object variable, and the age at surgery, preoperative CEA, preoperative MP, and preoperative AI as the explanatory variables. A receiver operating characteristic (ROC) curve was constructed to obtain the optimal cut-off value for preoperative radiographic parameters to distinguish between good and poor outcomes. Intraobserver and interobserver reliabilities for the measurement of radiographic parameters were investigated using the interclass correlation coefficient (ICC). All statistical analyses were performed using SPSS, version 23 (SPSS Inc, Chicago, IL), and the significance level was set at *P* ≤ .05.

## 3 Results

### 3.1 Clinical outcomes in the entire study sample

**Figure 2:**
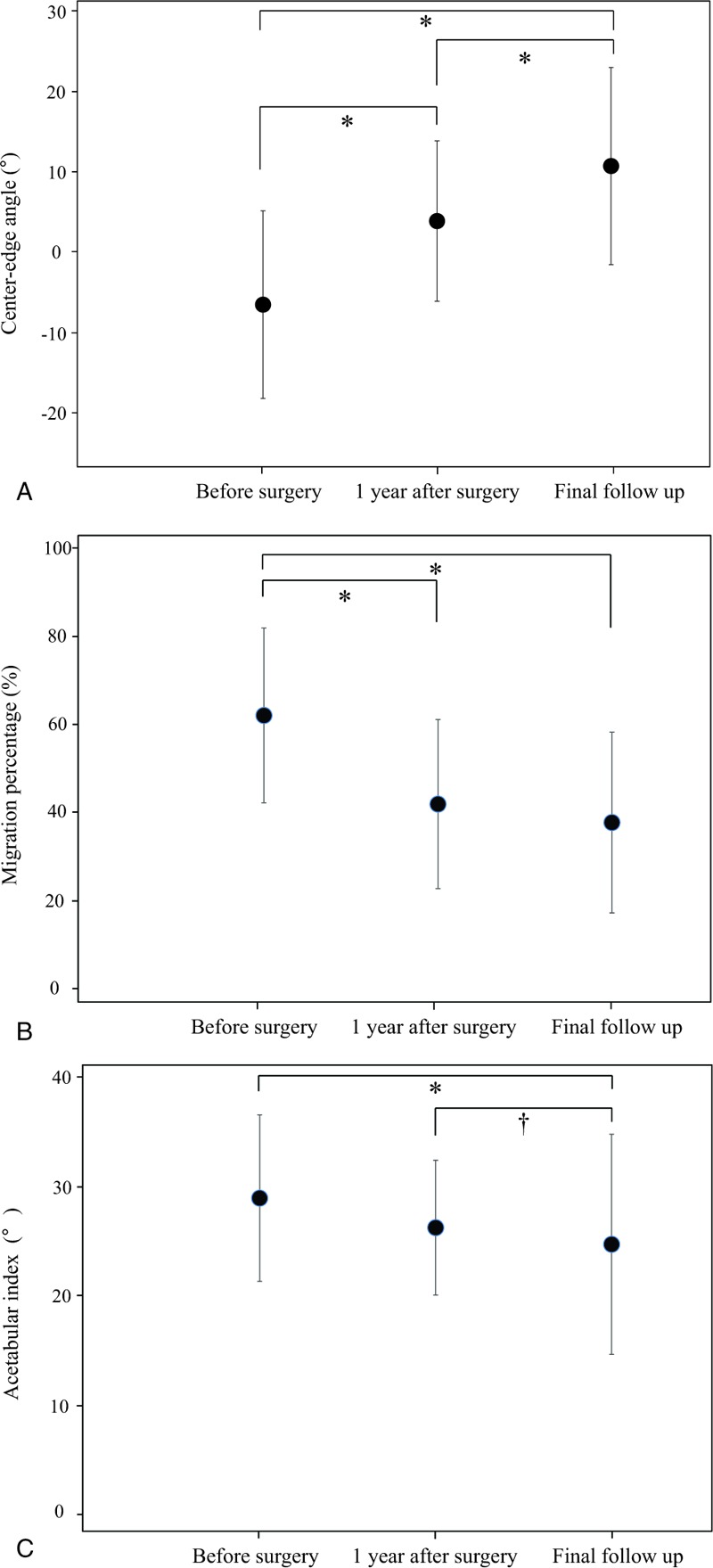
Comparison of radiological parameters using 1-way analysis of variance (ANOVA) test before surgery, 1 year after surgery, and final follow-up. A, Center-edge angle. B, Migration percentage. C, Acetabular index. Improvement can be seen over time with all parameters. Error bars indicate the average and standard deviation. ^∗^*P* < .01, ^†^*P* = .03.

All radiographic parameters were found to improve over time (Fig. 2A, B, and C). The mean CEA was −6.3° ± 11.7° preoperatively, 4.1° ± 9.9° at 1 year after surgery, and 10.9° ± 12.3° at final follow-up, with significant difference between the CEA values noted at 3 different time points (by 1-way ANOVA, *P* < .001). The mean MP was 62.0% ± 19.7% preoperatively, 42.0% ± 19.0% at 1 year after surgery, and 37.9% ± 20.4% at final follow-up, with a significant difference between the preoperative values and those noted at 1 year after surgery (*P* < .001), but no significant difference between the values noted at 1 year after surgery and at final follow-up (*P* = .24). The mean AI was 29.0° ± 7.5° preoperatively, 26.4° ± 6.1° at 1 year after surgery, and 24.9° ± 9.9° at final follow-up, with no significant difference between the preoperative values and those noted at 1 year after surgery (*P* = .09). A significant difference was, however, observed between the mean AI observed at 1 year after surgery as compared from that observed at final follow-up (*P* = .03). At final follow-up, we observed slightly improved gross motor function as 2 patients belonged to the GMFCS level III and another 2 patients to the level IV. The remaining 23 patients still belonged to the GMFCS level V. The outcome was good (MP < 33%) in 53% (23/43) of affected hips.

### 3.2 Prognostic factors

The present study has also revealed the radiological leading indicator predicting the clinical prognosis before the soft tissue release surgery. The preoperative values of the radiological parameters were compared between the groups of patients with good and poor outcome. These groups exhibited statistically significant differences in the preoperative CEA, MP, and AI values (*P* = .001, *P* = .003, and *P* = .001, respectively) irrespective of the age at the time of surgery (*P* = .40) (Table 2).

**Table 2:**
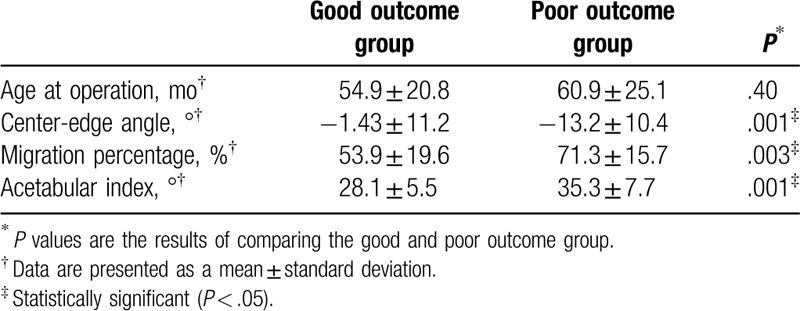
Comparison of preoperative radiographic parameters between the good outcome group and poor outcome group.

Multiple regression analysis was then performed to identify prognostic factors after soft tissue release surgery. Among various parameters we found only preoperative AI value exhibited the predictive value for the clinical prognosis after soft tissue release surgery (*P* = .01) (Table 3). However, the age at the time of surgery, preoperative CEA, or preoperative MP did not constitute as an independent predictor for clinical prognosis (*P* = .53, *P* = .15, *P* = .41).

**Table 3:**
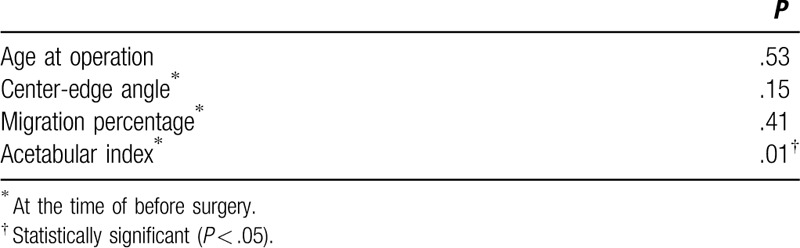
Results of multiple regression analysis.

**Figure 3:**
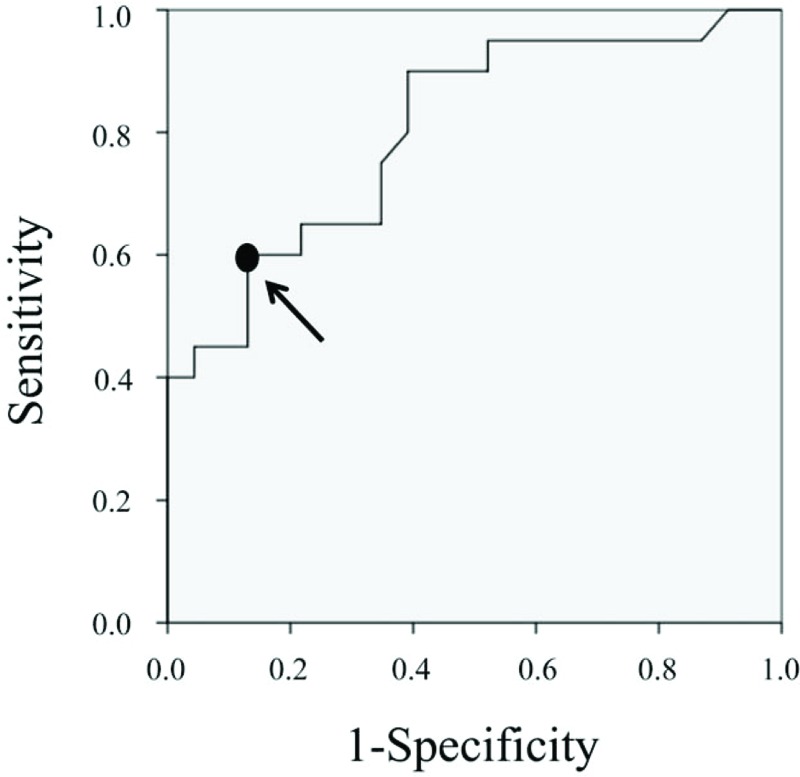
Receiver operating characteristics (ROC) curve based on each value of acetabular index (AI) on radiographic at the time of before surgery. We consider the data point 34° (arrow) on ROC curve as the cut-off value to divide the good outcome group and poor. Area under the curve (AUC) was 0.804.

Figure 3 shows the result of ROC curve analysis indicating that the optimal cut-off value of preoperative AI to differentiate between good and poor outcome was 34° with specificity of 87% and sensitivity of 60% (area under the curve of 0.804).

### 3.3 Reliability of radiographic measurements

The reliability of present radiographic assessment was evaluated by the variations of such assessment within the repeated measurement of the same investigator and the independent measurement by multiple investigators. The intraobserver and interobserver reliabilities thus evaluated for the measurement of radiographic parameters are summarized in Table 4. In addition, the ICCs for intraobserver and interobserver reliabilities were from 0.94 to 0.98 and from 0.91 to 0.97, respectively, indicating high reliabilities.

**Table 4:**
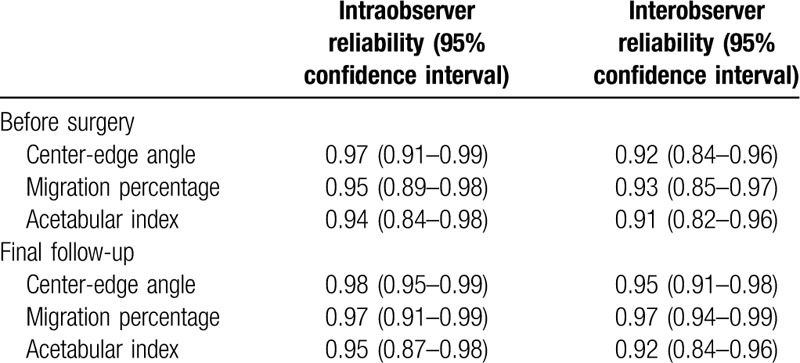
Reliability of x-ray parameters.

### 3.4 Representative cases

#### 3.4.1 Case 1

**Figure 4:**
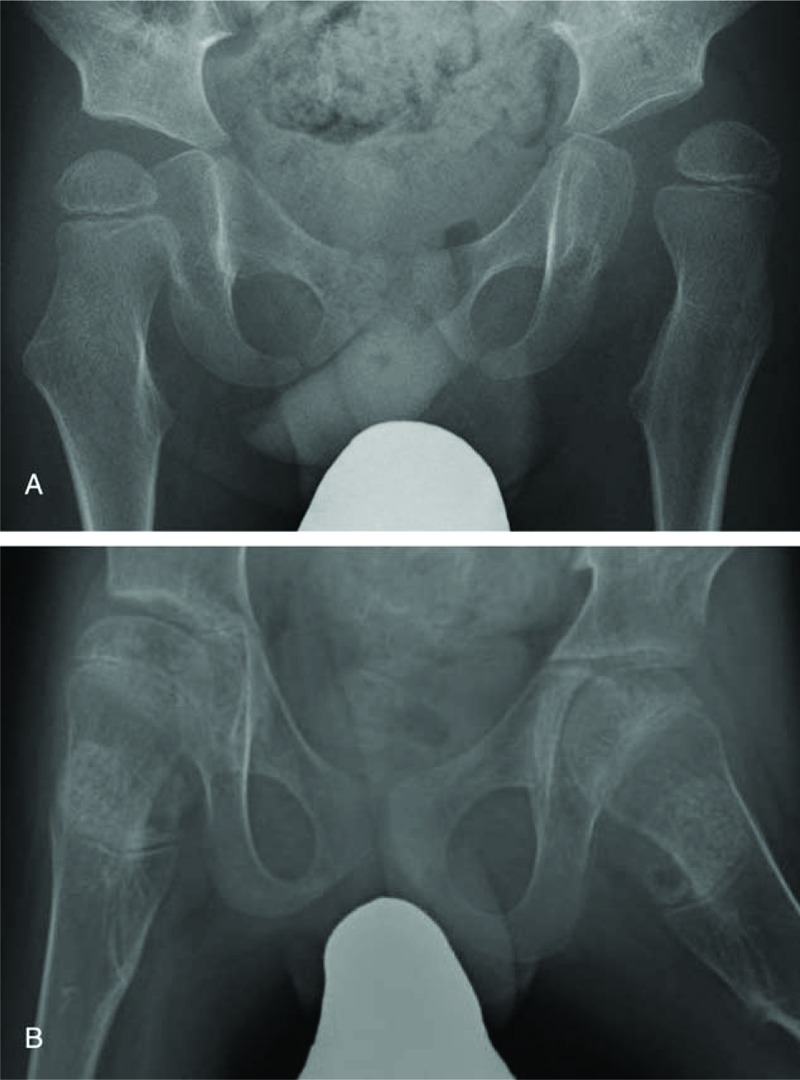
Radiographic representation of a patient with good prognosis before and after the soft tissue release surgery. Radiographs showing the hips of a boy with cerebral palsy, Gross Motor Function Classification System (GMFCS) level V. A, At 3 years of age with a migration percentage (MP) of 23% on the right side and 60% on the left side, acetabular index (AI) of 20.5° on the right side and 23.4° on the left side. B, 12 Years after soft tissue release, MP was 20% on the right side and 19% on the left side, with AI of 13.4° on the right side and 9.6° on the left side.

A boy with cerebral palsy, GMFCS level V, underwent soft tissue release surgery at 3 years of age. Before surgery, the MP was 23% on the right side and 60% on the left side, whereas the AI was 20.5° on the right side and 23.4° on the left side. At 12 years after surgery, the MP was 20% on the right side and 19% on the left side, with an AI of 13.4° on the right side and 9.6° on the left side (Fig. 4).

#### 3.4.2 Case 2

**Figure 5:**
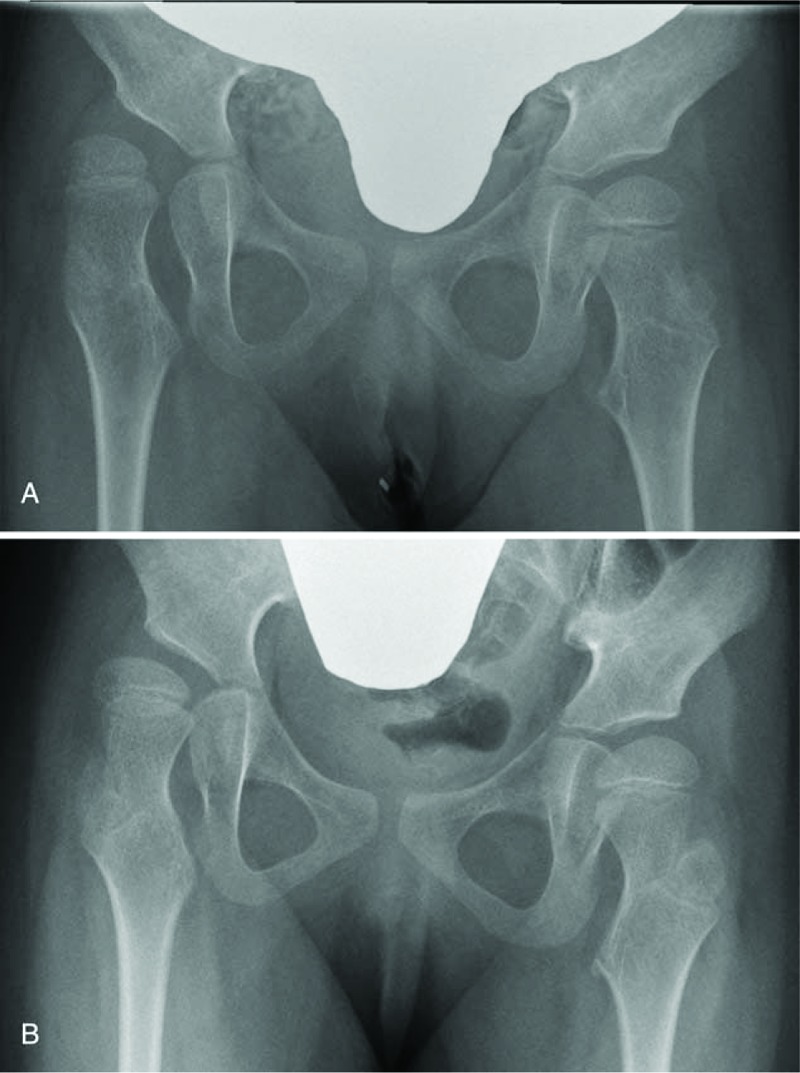
Radiographic representation of a patient with poor prognosis before and after the soft tissue release surgery. Radiographs showing the hips of a girl with cerebral palsy, Gross Motor Function Classification System (GMFCS) level V. A, At 3 years of age with a migration percentage (MP) of 62% on the right side and 24% on the left side, acetabular index (AI) of 37.1° on the right side and 20.6° on the left side. B, 3 Years after soft tissue release, MP was 41% on the right side and 26.7% on the left side, with AI of 35.3° on the right side and 18.3° on the left side.

A girl with cerebral palsy, GMFCS level V, underwent soft tissue release surgery at 3 years of age. Before surgery, the MP was 62% on the right side and 24% on the left side, whereas the AI was 37.1° on the right side and 20.6° on the left side. At 3 years after surgery, the MP was 41% on the right side and 26.7% on the left side, with an AI of 35.3° on the right side and 18.3° on the left side (Fig. 5).

## 4 Discussion

In this study involving 27 children with cerebral palsy and paralytic hip subluxation (MP ≥ 33%), soft tissue release surgery has improved the CEA from average −6.3° to 10.9°, the MP from average 62.0% to 37.9%, and the AI from average 29.0° to 24.8° over the course of an average follow-up period of 65 months. The outcome was good in 53% (23/43) of affected hips, which is within the range of previously reported success rates (33%–90%) following soft tissue release surgery for paralytic hip subluxation.^[7,15,16]^ This wide range of reported success rates might be due to differences among the studied patient population, especially regarding the degree of neurological involvement, age at surgery, indication of surgery, preoperative radiographic parameters evaluated, surgical techniques, postoperative cares, gross motor function (GMFCS level), definitions of operative success, and duration of follow-up.

Focusing on the problem of the functional capacity with regards to ambulation and performance of ADLs in this study, 25 patients belonged to the level V category in accordance with the preoperative GMFCS, one each patient belonged to the level III and IV among the 27 patients. At final follow-up, we observed improved gross motor function as 2 patients; 1 patient improved to level III from preoperative level IV, and another one improved to level IV from preoperative level V. Therefore, in total at final follow-up, 2 patients of 27 children belonged to level III and another 2 patients to the level IV, whereas the remaining 23 patients still belonged to the level V. Although there was a limit from the small sample size, we speculated the soft tissue release surgery for preventing paralytic hip subluxation in cerebral palsy might have some significance for preventing the exacerbation of exercise capacity.

The surgical indication for soft tissue release surgery is typically based on several factors such as the extent of displacement of the femoral head or the presence of acetabular dysplasia. It is highly desirable to determine the surgical indication in consideration of appropriate prognostic factors. Therefore, in this study, we evaluated several potential prognostic factors of soft tissue release surgery for managing hip subluxation, including age at the time of surgery and preoperative MP, CEA, and AI.

Scrutton et al^[17]^ enrolled 346 children with bilateral cerebral palsy born between 1989 and 1992 and followed-up until the age of 5 years. They concluded that, when measured correctly, the MP was the best indicator for monitoring hip subluxation as well as ascertaining the need for treatment. Bishay^[18]^ enrolled 50 children (100 hips) with a mean age of 3.6 years. He reported that children with higher preoperative MP exhibited less favorable outcome than those of lower preoperative MP, suggesting that early detection and application of the above-described treatment strategy should provide satisfactory outcomes in children with spastic hip disease.

Reimers^[7]^ demonstrated that, after hip subluxation occurs, there is a risk of a 10% increase in the MP per year. He also stated that spontaneous improvement of a subluxated hip cannot be expected, and suggested that bilateral adductor tenotomy should be performed as soon as hip subluxation is noted to obtain the good long-term outcomes in patients with MP > 33%. In our present study, we also noted that preoperative MP was significantly smaller in patients with good outcome than in those with poor outcome. We also agree that it is favorable to perform soft tissue release surgery, while the MP is still relatively low. The MP is, however, strongly influenced by the posture of the hip joints and the muscle tonus at the time of radiographic measurement. The same is true for the CEA, suggesting that neither the MP nor the CEA may be the only radiographic parameter to be considered upon soft tissue release surgery, as these parameters may not always be evaluated accurately.

Onimus et al^[15]^ enrolled 24 children with total-body-involved cerebral palsy and successful outcomes were obtained in 90% of patients younger than 4 years of age who had an MP <33%. Thus, they suggested that preventive surgery should be performed at 2 or 3 years of age, before the onset of hip dysplasia. Vidal et al^[19]^ assessed the clinical and radiological outcomes of the surgical correction of muscle imbalance in 43 children younger than 4 years at the time of surgery, and 63 children older than 7 years at the time of surgery. The authors found that the improvement was twice as large in the younger group than in the older group. In the present study, the age at the time of surgery did not differ significantly between children with good outcome and those with poor outcome (Table 2). Reimers^[7]^ suggested that, once hip subluxation occurs, the MP may increase by 10% each year. Therefore, the higher rate of success among patients with lower age at the time of surgery might be related to the fact that, after a certain threshold (eg, 33%), MP increases with age. In the present study, our surgical strategy prevented hip dislocation or substantial subluxation in 53% (23/43) of hips in patients followed up for a mean of 5 years and 5 months. This rate of success is not as high as other values reported in the literature, which may be related to less favorable preoperative gross motor function (less favorable distribution of GMFCS levels) of our patients. Several studies indicated that better preoperative gross motor function was associated with better outcomes after soft tissue release surgery.^[20–22]^ In the present study, 25 out of 27 patients had very poor gross motor function preoperatively, with GMFCS level V.

Cooke et al^[23]^ enrolled 332 patients and evaluated hip dislocation on 1684 radiographs of the pelvis and hip, as well as abdominal or spinal radiographs that included the pelvis. They concluded that the AI, accounting for the rotation of the acetabulum, was the most powerful predictor of hip dislocation. Cornell et al^[24]^ reviewed 56 hips in 37 children with cerebral palsy. In their study, surgery was unsuccessful in 13 out of 15 hips with an AI of >27°, and children with AI < 27° exhibited better outcomes after soft tissue surgery alone, although the difference was not statistically significant. In the present study, multiple regression analysis has revealed that only AI was an independent factor determining the prognosis of soft tissue release surgery for preventing paralytic hip subluxation in children with cerebral palsy. ROC curve analysis showed that the optimal cut-off value of preoperative AI for distinguishing good and poor outcome is 34°. That is, good outcome can be expected with soft tissue release surgery in patients with AI < 34°, whereas additional osseous surgeries might be necessary to achieve good outcome in patients with AI ≥ 34°. Since the AI is hardly influenced by the posture of the hip joint and muscle tonus, as is the case with the MP and CEA, where great care should be exerted when setting up the radiographic examination, to ensure adequate posture of the patient's hip. Based on our present results, we conclude that the AI is useful as a prognostic factor to be considered when determining the surgical indication of soft tissue release surgery for preventing paralytic hip subluxation in children with cerebral palsy.

The present study had several limitations, however, such as short follow-up, small sample size, and lack of a control group. In addition, muscle tonus was not evaluated and therefore the analyses were not controlled for the influence of muscle tonus on the relationship between preoperative radiographic parameters and the outcome of soft tissue release surgery. Therefore, further studies are needed to determine the precise indications of soft tissue release surgery for preventing paralytic hip subluxation in children with cerebral palsy.

## 5 Conclusions

The results of the ROC curve analysis indicate that the outcome of soft tissue release surgery to prevent severe paralytic hip subluxation is strongly influenced by the preoperative AI value. We found a statistically significant radiographic parameter in predicting better outcomes for patients under consideration of soft tissue release surgery. Specifically, those patients with lower preoperative AI (<34°) of the affected hip joint exhibited significantly better probability in preventing hip subluxation. Thus, the AI serves as a helpful parameter to consider soft tissue release surgery for children with cerebral palsy in preventing severe paralytic subluxation and dislocation.

## Author contributions

**Conceptualization:** Myongsu Ha.

**Data curation:** Myongsu Ha, Toshitsugu Fukuta, Yoshiaki Tsuboi, Yasuhiro Shirai, Kazuki Hattori, Eisuke Sakuma.

**Formal analysis:** Myongsu Ha, Takashi Okamoto, Kazuki Hattori, Eisuke Sakuma.

**Investigation:** Myongsu Ha, Toshitsugu Fukuta, Yoshiaki Tsuboi, Yasuhiro Shirai, Kazuki Hattori, Eisuke Sakuma.

**Project administration:** Myongsu Ha.

**Supervision:** Takashi Okamoto, Kenjiro Wakabayashi, Ikuo Wada, Takanobu Otsuka.

**Writing – original draft:** Myongsu Ha.

**Writing – review and editing:** Takashi Okamoto, Eisuke Sakuma, Kenjiro Wakabayashi, Ikuo Wada, Takanobu Otsuka.
